# Reviewed article: Lopes LPN, Raimundo ACS, Caetano R, Lopes LC. Recalculation of the budgetary impact of risperidone use for Autism Spectrum Disorder in Brazil: a case study using measured demand data, 2017-2019. Epidemiol Serv Saude. 2025:34;e20240732.

**DOI:** 10.1590/S2237-96222025v34e20240732.c

**Published:** 2025-08-08

**Authors:** Glaucio de Oliveira Nangino

**Affiliations:** Unimed Belo Horizonte, Atenção Domiciliar

**Table:** 

Rating	Excellent	Good	Average	Below average	Poor
Interest	✓				
Quality	✓				
Originality		✓			
Overall	✓				

**Table:** 

Questionnaire	Yes	No	Not applicable
Does the manuscript contain new and significant information to justify publication?	✓		
Does the Abstract (Summary) clearly and accurately describe the content of the article?	✓		
Is the problem significant and concisely stated?	✓		
Are the methods described comprehensively?	✓		
Are the interpretations and conclusions justified by the results?	✓		
Is adequate reference made to other work in the field?	✓		

## Manuscript Structure

Length of article is: Adequate

Number of tables is: Adequate

Number of figures is: Adequate

Please state any conflict(s) of interest that you have in relation to the review of this paper (state “none” if this is not applicable): None

## Comments

A avaliação crítica da fragilidade metodológica dos estudos prévios de viabilidade para a incorporação de tecnologias pelo SUS é o grande mérito do seu artigo. Entendo que seria de grande valor para o gestor que mais estudos de acompanhamento do impacto econômico após a incorporação de tecnologias possa ajudar até mesmo a calibração das estimativas futuras. Entendo que seria também desejável o acompanhamento da eficácia clínica dessas incorporações, a fim de comparar os estudos originais da literatura com a sua aplicação no contexto brasileiro, com todas as suas particularidades. 

